# Essential Tremor Suppression with a Novel Anti‐Tremor Orthosis: A Randomized Crossover Trial

**DOI:** 10.1002/mds.30082

**Published:** 2025-01-21

**Authors:** Winfred Mugge, Liset E.M. Elstgeest, Milan van Ginkel, Lucas Pol, IJsbrand de Lange, Nicola Pambakian, Alvaro Assis de Souza, Rick C. Helmich, Daan J. Kamphuis

**Affiliations:** ^1^ Faculty of Mechanical Engineering, Department of Biomechanical Engineering Delft University of Technology Delft The Netherlands; ^2^ Reinier de Graaf Hospital, Reinier Academy Delft The Netherlands; ^3^ Department of Neurology Reinier de Graaf Hospital Delft The Netherlands; ^4^ Department of Research and Development STIL B.V Delft The Netherlands; ^5^ Donders Institute for Brain, Cognition and Behaviour, Centre of Expertise for Parkinson and Movement Disorders, Department of Neurology Radboud University Medical Centre Nijmegen The Netherlands

**Keywords:** anti‐tremor, biomechanical loading, clinical investigation, essential tremor, mechanical joint damping, medical device, non‐invasive device, orthosis, treatment, tremor reduction, tremor suppression

## Abstract

**Background:**

Essential tremor (ET) is characterized by action tremor of the arms, which can interfere substantially with daily activities. Pharmacotherapy may be ineffective or associated with side effects, and stereotactic surgery is invasive. Hence, new accessible treatment options are urgently needed. An easy‐to‐use and lightweight orthotic device that exerts joint damping may provide an alternative solution for reducing tremor in daily activities.

**Objective:**

Our goal was to assess the efficacy of a novel anti‐tremor orthosis (STIL) in reducing clinical and accelerometry measures of distal arm tremor in ET.

**Methods:**

In a randomized crossover single‐blinded trial in 24 ET patients in a hospital setting, we compared three conditions: no orthosis (baseline), a sham device, and the anti‐tremor orthosis (order randomized). The orthosis, but not the sham device, passively damped joints in the forearm. Participants performed seven tasks from the Tremor Research Group Essential Tremor Rating Scale (TETRAS). The two co‐primary outcome measures were: clinical tremor severity (video‐scored TETRAS) and tremor power (accelerometry). Patient satisfaction was self‐assessed using the Dutch Quebec User Evaluation of Satisfaction with assistive Technology. Conditions were compared using Wilcoxon signed‐rank tests.

**Results:**

The anti‐tremor orthosis significantly reduced TETRAS scores compared to sham and baseline (baseline: 19.0 ± 3.2, sham: 13.7 ± 3.9, orthosis: 9.9 ± 3.6; mean ± standard deviation). Similar effects were observed for tremor power, which was reduced by 87.4% (orthosis vs. baseline) and 59.5% (orthosis vs. sham) across all tasks. A total of 71% of participants were (very) satisfied and 12.5% reported minor adverse events (discomfort/redness of skin).

**Conclusion:**

The anti‐tremor orthosis had a clinically relevant tremor‐reducing effect in ET in a controlled setting, offering potential for a new treatment to manage ET in daily activities. © 2025 The Author(s). *Movement Disorders* published by Wiley Periodicals LLC on behalf of International Parkinson and Movement Disorder Society.

## Graphical abstract

A novel anti‐tremor orthosis was evaluated in 24 essential tremor patients in a randomized crossover trial. It significantly reduced tremor severity and power in ADL and posture tasks compared to baseline and a sham device. The orthosis showed high patient satisfaction, offering a promising new treatment option for managing tremor.

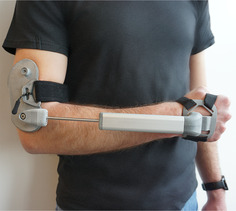

Tremor is an involuntary and rhythmic movement of a body part,^1^ which is often seen as a symptom in Parkinson's disease, dystonia, and essential tremor (ET).^2^ ET is the most common neurological movement disorder, affecting approximately 5% to 6% of the population over 65 years of age.^3^ The pathophysiology of ET has been related to abnormal oscillatory activity in the cerebellum, which is transmitted to the cerebello‐thalamo‐cortical loop.^4^, ^5^, ^6^ ET is characterized by a (bilateral) action tremor in the arms, occurring either during voluntary movement (kinetic tremor) or when the limb is held against gravity (postural tremor).^7^, ^8^ As a result, approximately 75% of people diagnosed with ET experience difficulties in performing activities of daily living (ADL), such as eating, drinking, and writing.^9^ Alongside the functional limitations, ET patients commonly experience feelings of shame related to their inability to engage in social activities.^10^

ET is most commonly treated with medication, and most evidence exists for propranolol, primidone, and topiramate,^11^ which may reduce on average 54% to 59% of the tremor amplitude, but can also introduce side effects such as dizziness, confusion, bradycardia, and fatigue.^12^ Up to 53% of patients discontinue pharmacological treatment because of side effects or lack of efficacy.^13^, ^14^ If medication is ineffective, stereotactic surgery can be considered, for example, deep brain stimulation (DBS) of the ventral intermediate nucleus (VIM) or subthalamic zone. DBS is highly effective in reducing tremor (45%–85%),^15^ but is also invasive and can lead to side effects,^11^ ranging from paresthesia and dysarthria to gait and balance disturbances.^16^, ^17^ Its efficacy on the long‐term cannot always be accurately predicted.^18^ Lesioning of the VIM through γ knife thalamotomy (GKT) or high‐intensity focused ultrasound (HIFU) also has an increasing role in the treatment of ET, reducing tremor severity up to 54% and 47%, respectively,^19^, ^20^ but are not widely available and are also invasive.

There has recently been an increased interest in wearable devices to reduce tremor.^21^ For instance, transcutaneous afferent patterned stimulation (TAPS) applied to the median and radial nerve reduced tremor power by ≥50% in 54% of patients.^22^ In a small ET cohort, an orthosis that biomechanically interferes with the arm dynamics reduced tremor power up to 81%.^23^ For such orthotic devices, good usability combined with effective tremor suppression remains a challenge, which has prevented its widespread use, especially among older people.^24^

Here, we test the effectiveness of a novel anti‐tremor orthosis developed by STIL B.V.. According to usability tests where users (n = 10) periodically wore the orthosis in home setting over the course of 2 days, the device (315 g) was found to be easy to don and doff and still allowed full freedom of movement in the arm while wearing the device. The orthosis contains biomechanical dampers that act on wrist flexion‐extension and forearm pronation‐supination movements, which are the joints contributing most to tremor power in ET.^25^, ^26^ In this cross‐over clinical trial, we contrasted the effect of the anti‐tremor orthosis with a sham device (without biomechanical dampers), and with baseline (no orthosis) on clinical tremor severity (Tremor Research Group Essential Tremor Rating Scale [TETRAS]) and tremor power (accelerometry). Last, we assessed patient satisfaction and monitored adverse events.

## Methods

### Study Design and Procedure

We performed a single‐blind, crossover randomized controlled trial to evaluate the STIL anti‐tremor orthosis on distal action tremor in ET patients. All participants were diagnosed with ET by Dutch neurologists, according to national guidelines,^27^ which are based on the 2018 Movement Disorder Society (MDS) consensus statement.^1^ This study was registered as clinical trial (isrctn.com: ISRCTN17323638) and approved by the local medical ethics committee (NL79108.000.21). All participants provided informed consent.

#### Patient Selection

Participants were recruited for the study in the Reinier de Graaf Hospital (Delft, The Netherlands); inclusion criteria included: age (>18 years); difficulty with ADL (Bain and Findley^28^ >30 of 72 from 14 items with a max score of 4 and doubling the scores of the first four items to add weight to upper extremity tasks); tremor severity (TETRAS^29^ score >13 on subset of 3 ADL and 4 upper extremity tasks); and the presence of primarily distal action tremor in either of the arms. Additionally, participants needed to have a stable dose of tremor‐reducing medication for at least 30 days (90 days for anti‐depressants) before enrolment, and were required to abstain from consuming coffee or alcohol for 10 hours and avoid heavy physical exercise for 24 hours before the interventions. Exclusion criteria for participants were: diagnosis of diseases or disorders other than ET that feature tremor as a symptom (eg, functional tremor, multiple sclerosis, peripheral neuropathy, Parkinson's disease); diagnosis of epilepsy or dementia; primarily shoulder internal‐external rotation tremor and/or elbow flexion‐extension tremor; use of medication known to exacerbate tremor; prior stereotactic surgery for tremor; botulinum toxin injections in the arm <6 months before enrolment; restricted muscle function (eg, contractures or spasticity); severe head tremor (to avoid interference with scoring tasks such as eating and drinking); damaged skin or infections on the location were the orthosis is worn; inability to fit the orthosis because of arm size (upper arm circumference >350 mm); inability to understand the study procedures; history of excessive alcohol consumption; and pregnancy.

### Procedure

Eligible participants visited the hospital for a 2‐hour appointment. We measured tremor severity and power in three conditions: wearing no orthosis (baseline), wearing a sham orthosis (sham), and wearing the anti‐tremor orthosis (orthosis). Only one arm was evaluated. We always started with the baseline condition, which was followed by sham and orthosis (order randomized). In each of the three cases, patients performed a pre‐specified subset of seven tasks derived from the TETRAS: three movements (static posture of both arms, wingbeat posture, and finger‐to‐nose) and four ADL tasks (spiral drawing, pouring, drinking, and eating). Participants were requested to perform each of these tasks for 30 seconds while being video recorded. Simultaneously, motion data was recorded at 100 Hz from an inertial measurement unit (IMU) sensor (Xsens Technologies B.V., MTw Awinda, The Netherlands) strapped to the participant's proximal phalanxes of both the index and middle finger. The IMU was kept in place when swapping between the orthosis and the sham. Participants were told they would be testing two variants of the orthosis, not that one was a sham. The devices were donned by the investigator, to prevent participants from inspecting individual joints and comparing the devices. Last, participants were unaware of the order in which the devices were tested (sham or orthosis) to ensure blinding.

Patient satisfaction with regards to comfort and usability of the devices was scored using the Dutch Quebec User Evaluation of Satisfaction with assistive Technology (D‐QUEST) questionnaire.^30^ D‐QUEST was assessed after the second and third conditions (ie, sham and orthosis). Last, adverse events were reported.

### Investigational Devices

Tremor can be reduced with the addition of joint damping.^23^ A systematic review mentioned several orthoses showing promise in passively reducing tremors.^31^ The novel anti‐tremor orthosis (STIL B.V., Delft, The Netherlands), is a non‐invasive device designed to reduce tremors by damping wrist flexion‐extension and forearm pronation‐supination, and restraining wrist radial‐ulnar deviation. Pigg et al^25^ identified that kinetic tremor power is most dominant in forearm pronation‐supination (FPS) and wrist flexion‐extension (WFE), followed by wrist radial‐ulnar deviation (WRUD) and shoulder internal‐external rotation (SIER).^25^ The distinguishing factor of the novel orthosis is that it stabilizes three joints (FPS, WFE, and WRUD) simultaneously, where other passive orthoses predominantly focused on single joints. A combination of both friction and viscous dampers for WFE and FPS limit high‐frequency involuntary movements (such as tremor) while minimizing interference with voluntary movements. The device physically limits WRUD. Damping‐coefficients were optimized empirically to find an optimal balance between movement reduction and freedom of movement. The device does not use sensors, (electromagnetic) actuators, or other electronics.

**FIG. 1:**
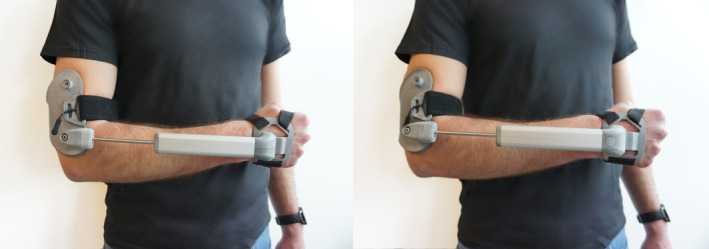
The anti‐tremor orthosis on the left, “sham” device on the right. The sham device has similar weight, usability, and feeling, but has no joint damping or a limit on wrist radial/ulnar deviation. The novel anti‐tremor orthosis consists of an elbow piece and a hand piece, connected by a rigid extension element, via a total of three joints. The orthosis is completely passive, and therefore, does not require software or electronics for its operation.

The sham device mimics the anti‐tremor orthosis in looks, user interface, and weight, but lacks dampers and permits motion in the WRUD joint (Fig. 1). Before this investigation, the sham was validated with users not to be perceived as sham according to a credibility and expectancy questionnaire (n = 16).

The addition of weight to the arm of ET patients is known to reduce tremor amplitude.^32^ Given that the sham adds weight to the arm (~315 g), it is expected to reduce tremor amplitude, but to a smaller extent than the orthosis. Evaluating tremor severity while wearing the sham serves to evaluate placebo effects on tremor reduction.

### Outcome Measures

Tremor severity was assessed by two neurologists specialized in movement disorders (R.C.H. and D.J.K.), who independently assessed the seven TETRAS tasks from video recordings, resulting in seven per‐task scores per participant per condition. In case two per‐task scores differed by more than 1 point, the neurologists' assessment was repeated in a joint session and results reevaluated. Finally, the two neurologists' per‐task scores were averaged. Raters were not blinded as the sham allowed visually observable radial‐ulnar deviation.

For the postures and ADL‐tasks, amplitude is rated peak‐to‐peak using TETRAS' upper limb tremor scale. The per‐task scores were averaged among all participants, to get a single per‐task score (eg, eating) for a specific condition (eg, sham). The total TETRAS score for a condition was obtained by summing all the per‐task scores, resulting in a maximum of 28 points (7 tasks, range 0–4).

Calculation of percentage reduction of the TETRAS score was achieved by using the logarithmic relationship between tremor amplitude and the clinical rating scale, as described by Elble et al,^33^ depicted in Equation 1. For the total score reduction, the value of α was calculated using the method from Elble et al,^16^ using Equation 2.

(1) $$\frac{T_{2}}{T_{1}\;}=10^{\alpha \;\left({\mathrm{TRS}}_{2}-{\mathrm{TRS}}_{1}\right)}$$

(2) $$\alpha_{s}=\alpha_{4}\left(\frac{4}{s}\right).$$

T_2_/T_1_ is the ratio of tremor amplitude reduction and TRS_2_‐TRS_1_ the difference in clinical rating score before and after an intervention. *α*_4_ is the α factor for a four‐point scale, being 0.355 for the TETRAS upper extremity tasks and 0.306 for the TETRAS ADL tasks.^33^ The total TETRAS score can be considered a 28‐point scale, and by using Equation 2, *α*_28_ is calculated to be 0.051.

Tremor power was measured from the hand accelerations as indicator of variations from the mean posture. Tri‐axial accelerometry readings (m/s^2^) were combined into a single vector by calculating the magnitude (vector magnitude = √(*x* ^2^ + *y* ^2^ + *z* ^2^)). This magnitude was then zero‐phase filtered using a second‐order Butterworth bandpass filter in the range 3 to 12 Hz using the *filtfilt* function (MATLAB 2021b, The MathWorks, Natick, MA), which is the frequency band in which ET typically occurs.^34^ This step removed any voluntary and gravitational components from the signal. Subsequently, the average bandpower (ie, the integral of the power spectral density) was calculated using MATLAB's *bandpower* function, to produce per‐task scores. Similar to the TETRAS score analysis, all per‐task scores per condition were summed to a total score. Tremor power reduction (in percentage) was calculated as one minus the ratio between compared conditions, times 100.

Patient satisfaction with the device was self‐assessed by the patients using the D‐QUEST. Only the first eight items of the D‐QUEST, relating to the device, were assessed; items about the service of the manufacturer were not. The answer categories on this scale ranged from 1 (“completely unsatisfied”) to 5 (“very satisfied”). The safety of the device was assessed from the listed adverse events, which were monitored by the investigator during the study.

### Statistical Analyses

Statistical analyses were performed using SPSS Statistics (version 25.0.0.2, IBM, Armonk, NY) and Python (version 3.10.15) with libraries *scipy* (version 1.12.0) for Wilcoxon tests and Spearman correlations, and *statsmodels* (version 0.14.1) for the false discovery rate (FDR) correction. All tests were 2‐sided with an α level of 0.05.

The total scores, as well as per‐task scores, were compared across two scenarios: orthosis versus baseline and orthosis versus sham. The differences in these scores constituted co‐primary outcome measures. A non‐significant difference between the sham and orthosis conditions may suggest the presence of placebo effects.

Because some per‐task scores of the tremor severity (ie, TETRAS scores) and tremor power (ie, accelerometry data) were not normally distributed, Wilcoxon signed rank tests were used to analyze differences in tremor severity and power over these comparisons. To correct for multiple comparisons, the Benjamini‐Hochberg correction was used for both analyses.

As an additional analysis, a principal component analysis (PCA) was performed based on tremor power, comparing groups including randomization order (sham‐orthosis vs. orthosis‐sham), sex and medication use. A PCA is a dimensionality reduction method that summarizes the available data (ie, accelerometry measurements) into variables known as principal components. By mapping the data points onto the space defined by the first two principal components and color‐coding them according to different categories of interest, we could identify patterns related to tremor power within the dataset. A power transformation was applied to the data before the PCA using default values.^35^ This transformation helps making the data more Gaussian‐like, also allowing a better visual understanding of the variability in the data.

To evaluate if both outcome measures show similar trends, a Spearman correlation was used for assessing the correlation between reductions in (inverse log‐transformed) tremor severity and tremor power. The *P*‐values for the correlations were obtained via permutation tests because of the small sample size. An α of 0.355 was used for correcting TETRAS score using Equation 1.^33^

For each D‐QUEST item, the mean and standard deviation (SD) were calculated. Difference between the orthosis and sham device were analyzed by Wilcoxon signed rank test.

## Results

### Participants

**FIG. 2:**
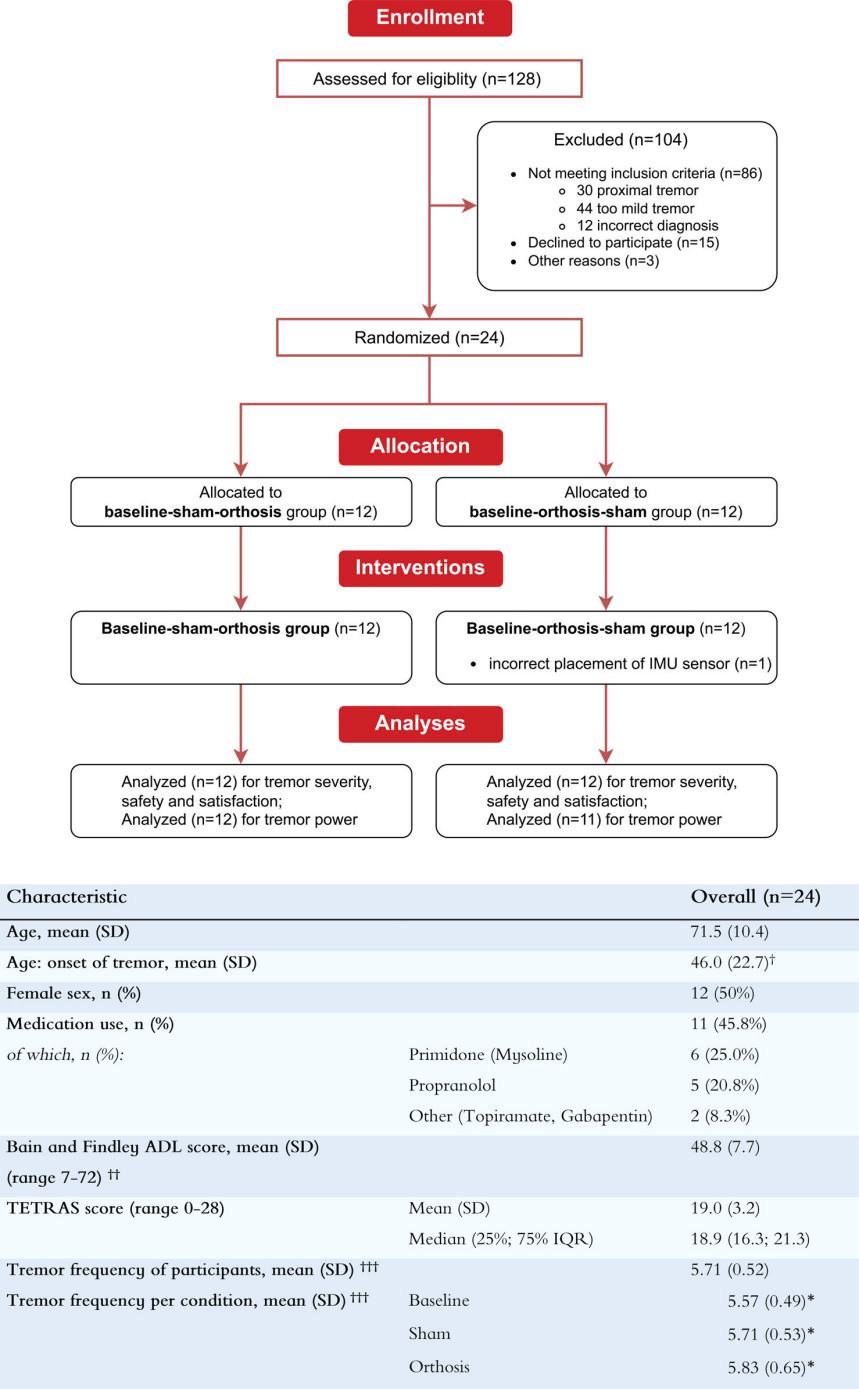
Consolidated Standards of Reporting Trials diagram showing the numbers of patient enrolment, allocation, and analysis (top) and descriptive analysis of the included participants (n = 24) (bottom). Of the 86 patients excluded, 30 patients (also) had a proximal arm tremor, for 44 patients the tremor was too mild and 12 patients had an incorrect diagnosis. ^†^For one participant, onset age was not known. ^††^Scores to items 1–4 of the Bain and Findley scale were doubled (maximum score 8), adding weight to upper extremity tasks. Therefore, a maximum total score of 72 point could be obtained. ^†††^First, peak frequency of each task was calculated from the band‐passed accelerometry signal, by finding the maximum value of the Fourier‐transformed data. Next, the median was calculated from all peak frequencies, and subsequently averaged, to get a mean tremor frequency per condition and for all participants. *Means of between conditions are not significantly different (*P* = 0.298, one‐way analysis of variance, *α* = 0.05). Table S1 contains extended patient demographics. [Color figure can be viewed at wileyonlinelibrary.com]

Based on a power analysis with effect size 0.8 (G*power, Universität Düsseldorf, Germany), a total of 24 participants completed the investigation (Fig. 2), out of whom one subject had an incorrectly placed IMU sensor, leaving 23 participants for the analysis of tremor power. Figure 2 also shows the characteristics of the study sample: mean (SD) age was 71.5 (10.4) years, whereas mean (SD) age of onset was 46.0 (22.7) years, 50% were female and 42% used medication for the tremor.

### Tremor Reduction per Group

Total scores of both tremor severity and tremor power significantly differed between conditions, both when comparing orthosis versus baseline and when comparing orthosis versus sham (Table 1). The difference between orthosis versus baseline (9.1 points reduction on 7 TETRAS items) was higher than orthosis versus sham (3.8 points reduction). Overall inter‐rater reliability (intraclass correlation coefficient) on the TETRAS scores over all three conditions between the two raters was good to excellent (0.88).

**TABLE 1:** Tremor severity and tremor power per condition and reductions per comparison

	Baseline	Sham	Orthosis	Tremor reduction (orthosis vs. baseline)		Tremor reduction (orthosis vs. sham)	
	Mean (SD)	Mean (SD)	Mean (SD)	Δ Mean	Reduction, %	Δ Mean	Reduction, %
TETRAS‐score (n = 24) range (0–28)							
**Total score**	**19.0 (3.2)**	**13.7 (3.9)**	**9.9 (3.6)**	−9.1***	**−65.4**	−3.8***	**−35.8**
Outstretched	2.1 (0.6)	1.4 (0.8)	0.9 (0.7)	−1.3***	−65.4	−0.6***	−38.8
Wing‐beating	2.2 (0.5)	1.6 (0.5)	1.1 (0.6)	−1.1***	−59.3	−0.5***	−33.5
Finger‐to‐nose	2.4 (0.3)	2.0 (0.4)	1.4 (0.6)	−1.0***	−50.6	−0.6***	−34.5
Spiral drawing	2.9 (0.7)	2.2 (0.7)	1.8 (0.6)	−1.2***	−57.1	−0.4**	−24.6
Pouring	3.0 (0.7)	2.1 (1.1)	1.4 (0.8)	−1.6***	−67.6	−0.7***	−38.9
Drinking	2.9 (0.8)	2.1 (0.8)	1.6 (0.7)	−1.4***	−62.7	−0.6***	−34.5
Eating	3.4 (0.8)	2.2 (0.8)	1.7 (0.7)	−1.6***	−67.6	−0.5**	−29.7
Tremor power (n = 23) (m/s^2^)^2^							
**Total score**	**65.5 (55.4)**	**20.4 (28.1)**	**8.3 (11.7)**	−57.3***	**−87.4**	−12.2***	**−59.5**
Outstretched	14.0 (29.8)	2.6 (7.9)	0.6 (1.8)	−13.4***	−95.8	−2.0*	−77.2
Wing‐beating	9.5 (17.4)	2.1 (7.9)	1.2 (4.8)	−8.4***	−87.6	−0.9**	−44.5
Finger‐to‐nose	7.8 (8.1)	2.7 (2.9)	1.5 (1.5)	−6.3***	−80.7	−1.2***	−44.7
Spiral drawing	6.6 (5.3)	2.0 (2.0)	0.5 (0.4)	−6.2***	−92.8	−1.5***	−75.9
Pouring	11.1 (10.6)	5.1 (8.2)	1.6 (1.8)	−9.4***	−85.3	−3.5***	−68.1
Drinking	9.6 (8.0)	3.0 (4.0)	1.3 (2.0)	−8.4***	−86.6	−1.7**	−56.6
Eating	6.9 (6.2)	2.9 (3.9)	1.6 (1.6)	−5.3***	−76.8	−1.3*	−45.7

Baseline, sham and orthosis are presented in mean (SD). Tremor reduction for both comparisons (orthosis vs. baseline and orthosis vs. sham) are presented both in an absolute way (eg, points reduction) and a relative (ie, percentage reduction). FDR corrected *P*‐values of Wilcoxon signed rank test: ***P < 0.001, **P < 0.01, *P < 0.05.

Abbreviations: TETRAS, Tremor Research Group Essential Tremor Rating Scale; SD, standard deviation; FDR, false discovery rate.

For both primary outcome measures, the per‐task tremor reductions with the orthosis were all substantial and significant compared to baseline. TETRAS reduction was highest for eating and pouring (both 67.6%), followed by outstretched posture (65.4%). Tremor power was reduced most in outstretched posture (95.8%), followed by spiral drawing (92.8%) and wing‐beating (87.6%).

**FIG. 3:**
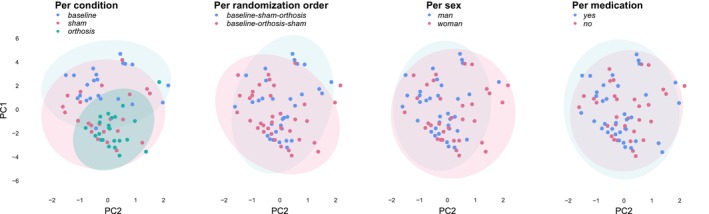
From left to right: results of the principal component analysis per condition, randomization order (sham‐orthosis vs. orthosis‐sham), sex and medication. Each dot represents an accelerometry data‐point from a participant in single condition (baseline, sham, and orthosis), scatter plotted against the two principal axes. [Color figure can be viewed at wileyonlinelibrary.com]

Figure 3 shows the PCA analysis, where we reduced the dimensionality of the bandpower of all tasks and all conditions into two principal components. The two components explained 71% and 10% of the total data variance, respectively. By color‐coding the group variables (per condition, randomization order, medication usage, and sex), we could identify the existence of patterns with respect to these principal axes. The only clear cluster is the “orthosis” on the per condition plot, indicating similar motion characteristics among this group.

### Tremor Reduction per Participant

**FIG. 4:**
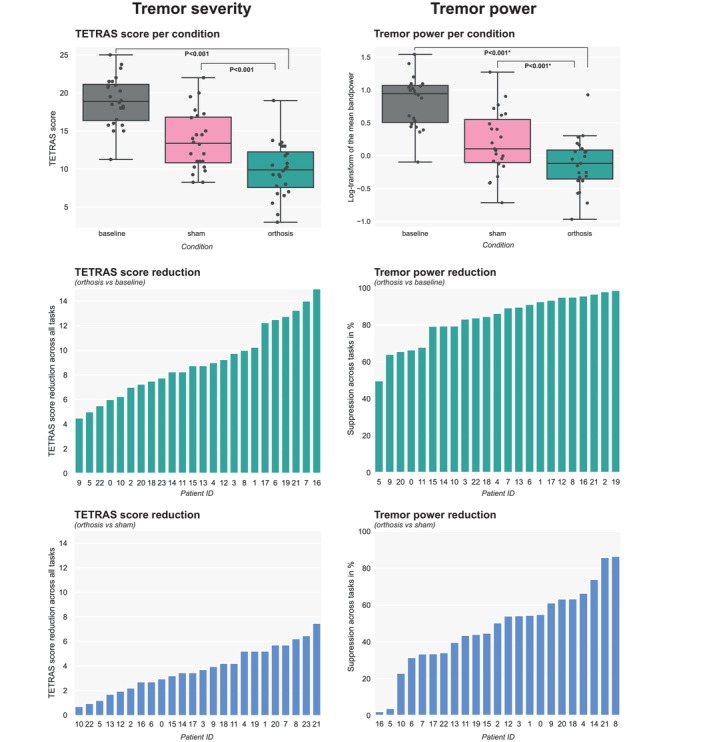
Patient‐specific outcomes. Top panels show reduction per condition, depicting the median and the interpatient spread. Middle panels show orthosis against baseline, bottom panels show orthosis against sham. Left panels show Tremor Research Group Essential Tremor Rating Scale (TETRAS) score reductions, right panels show tremor power reductions. The orthosis versus sham comparison depicts the devices' relative performance. With equal performing devices, the TETRAS score comparison would be zero, whereas for tremor power it would be 0%. *For visualization purposes the mean bandpower was log‐transformed for comparison with TETRAS scores, note that statistics was performed on the mean bandpower without log‐transform.

Figure 4 illustrates the tremor reduction per participant for both comparisons (orthosis vs. baseline and orthosis vs. sham). The inter‐patient spread is highest in the sham condition. For all participants, both the sham and orthosis had a tremor power and severity reduction. Spearman correlations between TETRAS score and tremor power were 0.71 (*P* = 0.0004) (orthosis) and 0.62 (*P* = 0.0018) (sham), indicating strong correlations.^36^ Figure S2 displays an accelerometry time trace for two participants in all three conditions. Figure S3 compares all participant's baseline tremor severity with orthosis' effectiveness.

### Patient Satisfaction and Adverse Events

Patient‐assessed general satisfaction showed that 71% of the participants were (very) satisfied (scores 4/5 and 5/5) with the orthosis (50% with the sham, *P* = 0.021). Note that “more or less satisfied” (score: 3/5) was not considered satisfied in this statistic. Mean (SD) patient‐assessed satisfaction with the effectiveness of the orthosis was 3.8 (0.9), compared to 3.5 (0.9) of the sham. Figure S1 shows the results of D‐QUEST. Three of 24 subjects had minor adverse events (discomfort of elbow area [n = 1], discomfort on dorsal side of the hand [n = 1], redness of the skin [n = 1]).

## Discussion

In this study, we evaluated a novel anti‐tremor orthosis for the suppression of action tremor of the forearm in 24 patients with ET. Our findings show a statistically significant and clinically relevant tremor reduction for all participants, both for clinical tremor severity and for tremor power. The measured tremor power reduction of 87% is comparable to the effects reached with conventional treatment options, and exceeds the observed effects for other commercially available wearable devices.^22^ A hand tremor reduction with a minimum of one TETRAS point has been shown as a satisfactory treatment effect for ET patients.^37^ As all of the seven tasks showed a significant decrease in TETRAS score of at least 1 point, the effect of the orthosis can be considered clinically relevant. Moreover, the 9.1 points reduction that we found would translate to 2.8 SD units of baseline condition, which substantially exceeds the 0.5 SD units considered to be a minimally clinically important difference (MCID).^38^ Secondary outcome measures showed that patients were satisfied with the device, and only minor adverse events were observed. This suggests that such non‐invasive orthotics could be used to complement existing treatments options for ET patients with a predominant distal action tremor of the arm.

The orthosis may reduce tremor through several mechanisms. The fact that the sham device also significantly reduced tremor, and the fact that tremor amplitude in ET is sensitive to added weight,^34^ suggests that loading may have played a role. However, the orthosis was significantly more effective (59.5% more tremor power reduction) than a sham device matched in weight, ruling out that this was the only contributing mechanism. Instead, mechanical damping to the joints of the forearm, which was present in the orthosis, but not the sham device, is likely the main contributor to the reduction of tremor severity/power by the orthosis. Tremorgenic cerebello‐thalamo‐cortical activity in ET may be modulated and exacerbated by oscillatory sensory input, and peripheral reflex loops also contribute to tremor.^4^, ^39^ For instance, limb perturbations have been shown to reset ET,^40^, ^41^ and closed‐loop median nerve stimulation influenced ET amplitude,^42^ suggesting that afferent inputs affect the central tremor oscillator. In line with this idea, thalamic deep brain recordings in ET showed that VIM oscillatory activity (derived from local field potentials) preceded bursting activity, suggesting that neuronal firing is entrained by periodic (tremor‐related) afferent inputs.^43^ Additionally, thalamo‐muscular coherence in ET occurred only after the onset of tremor, possibly because of somatosensory (muscle) feedback.^44^ Hence, beyond inducing a mechanical constraint, the orthosis might alter oscillatory sensory input and reflex loops in a manner that reduces tremorgenesis. Additionally, physical support provided by the orthosis might lead to decreased voluntary muscle contractions, which in turn can lead to lower tremor power.^45^ Additional research, for example, with electromyography and electroencephalography recordings, may help to further investigate such mechanisms. Finally, D‐QUEST questionnaire results show that there was no statistical difference in patient‐perceived effectiveness of the two devices, supporting the validity of the sham. Although the notable tremor reduction seen with the sham raises the possibility of some placebo effect related to wearing a device, the significantly greater tremor reduction with the orthosis versus sham indicates a real and substantial effect not because of placebo.

Strengths of this study are the sham‐controlled design, blinding and randomization of participants, and the inclusion of both examiner and quantitative instrumented outcome measures. There are also some limitations. First, only participants with diagnosed ET were included. Diagnoses were confirmed by experienced movement disorders neurologists (which excluded 12 patients during screening because of misdiagnosis), but misdiagnoses can still occur.^46^ Assessment of the TETRAS videos verified that all patients had a bilateral action tremor of the arms, without additional neurological signs (eg, dystonia, ataxia). However, no additional screening was done on the presence of ET‐plus. Additionally, although randomization groups were adequately balanced (Fig. 1) and no group biases are evident from the PCA, the study had a limited sample size. The intervention with the sham and orthosis lasted 1 to 2 hours per participant in a controlled hospital setting. Being a mechanical intervention, it is not expected that effectiveness of the orthosis would differ over time. However, long‐term effects of the orthosis in daily life and its psychosocial impact should be evaluated in further real‐world studies. Expert raters were unblinded to the sham and orthosis conditions. Despite strong correlation between TETRAS scores and accelerometry data, this may have introduced rater bias. Finally, we included ET patients with a mild to severe tremor, and excluded 74 patients with slight and/or proximal tremors (ie, 58% of the screened participants). Therefore, the orthosis may not be suitable for all (ET) patient groups. Moreover, action tremor also occurs in other tremor disorders, such as Parkinson's disease^47^, ^48^ and dystonia,^49^, ^50^ raising the possibility that joint dampening orthoses may be useful for these broader groups of patients.

## Conclusions

Using a single‐blind randomized crossover trial, executed in a controlled hospital setting, we showed that a novel anti‐tremor orthosis that mechanically dampens joint movements is effective in reducing tremor in ET patients with a distal action tremor, with only minor adverse events and high satisfaction scores. Our findings show that joint damping orthoses could be a valuable addition to the current treatment of ET.

## Financial Disclosures

This study was initiated and funded by the Reinier de Graaf Hospital. Investigational devices were loaned for the purpose of the study by STIL B.V. Development of the investigational devices was financially support by the Brain Foundation Netherlands (De Hersenstichting). IJ.d.L and N.P. are cofounders of STIL B.V., L.P. and A.A.d.S. work(ed) for STIL B.V., the company that developed the anti‐tremor orthosis. L.E.M.E., and M.v.G. have no financial disclosures. In the past 12 months, D.J.K. received fees from Abbvie for advisory board consultancy (on echoguided botulinum toxin injection therapy, s.c. levodopa treatment) and fees from Abbvie and Ipsen for courses (about echoguided botulinum toxin treatment); W.M. was supported by Health~Holland (top sector life sciences & health); R.C.H. was supported by the Michael J Fox Foundation and The Netherlands Organisation for Health Research and Development (ZonMw).

## Author Roles

W.M., L.E.M.E., R.H., IJ.d.L. and D.J.K. conceived the project; W.M., IJ.d.L., L.E.M.E., M.v.G., R.C.H., and D.J.K. organized it; and M.v.G., L.P., and D.J.K. executed it. Statistical analysis was designed by W.M., L.E.M.E., R.C.H., and D.J.K., executed by W.M., L.E.M.E., and A.A.d.S., and reviewed by R.C.H. and D.J.K. The manuscript was drafted by W.M., IJ.d.L., and A.A.d.S., with review by L.E.M.E., N.P., R.C.H., and D.J.K.

## Supporting information


**Figure S1.** Self‐reported satisfaction with devices (orthosis and sham) based on the D‐QUEST.

**Table S1.** Extended patient demographics.

**Figure S2.** Example data of accelerometry readings.

**Figure S3.** TETRAS baseline score VS. TETRAS reduction.

## Data Availability

The data is available for non‐commercial use via the Dutch national center of expertise and repository for research data (*DANS*) under restricted access, with the following DOI: https://doi.org/10.17026/LS/WV4ASD.
