# Supplementary material for: Essential Tremor Suppression with a Novel Anti‐Tremor Orthosis: A Randomized Crossover Trial
Source: Mov Disord. 2025 Jan 21;40(3):445–55. doi: 10.1002/mds.30082 (PMC11926495; doi:10.1002/mds.30082)
Supplement: Supplementary file 1 — Figure S1. Self‐reported satisfaction with devices (orthosis and sham) based on the D‐QUEST. [file MDS-40-445-s004.pdf]

D-QUEST Patient satisfaction of STIL orthosis

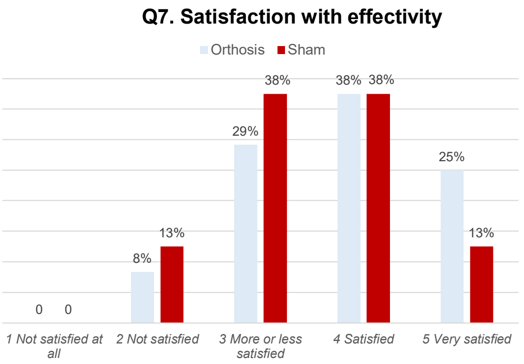

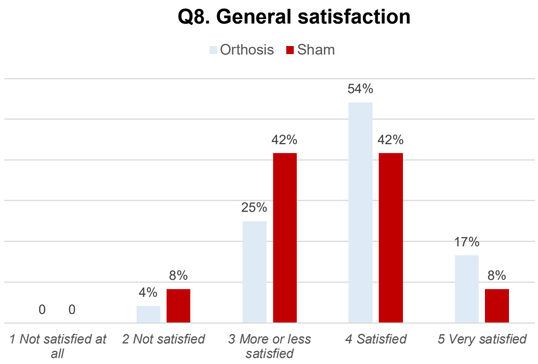

Question Category	Q1. Dimensions	Q2. Weight	Q3. Adjustability	Q4. Safety	Q5. Ease of use	Q6. Comfort	Q7. Effectiveness	Q8. Overall
Sham	3.7 (0.9)	4.1 (0.6)	3.8 (0.8)	4.0 (0.8)	3.8 (0.7)	3.6 (0.6)	3.5 (0.9)	3.5* (0.8)
Orthosis	3.4 (0.7)	3.8 (0.9)	3.8 (0.5)	3.9 (0.7)	3.5 (0.8)	3.3 (0.5)	3.8 (0.9)	3.8*(0.8)
Significance using Wilcoxon Signed Rank test*								*0.021**

Supplementary Figure S1: Self-reported satisfaction with the sham and orthosis after completing 7 tasks of the TETRAS scale. The table presents the 8 items of the D-QUEST (mean (SD), n=24). Range: 1 (Not satisfied at all), 2 (Not satisfied), 3 (More or less satisfied), 4 (Satisfied), 5 (Very satisfied). The graphs present item 7 (satisfaction with efficacy) and item 8 (general satisfaction). Participants were deemed satisfied when scores were equal or greater than 4. For both satisfaction with effectivity, as well as general satisfaction, participants were more satisfied with the orthosis in comparison to the sham: Q7 indicates 51% for sham versus 63% for orthosis, whereas Q8 has 50% for sham versus 71% for orthosis.
