# Supplementary material for: Essential Tremor Suppression with a Novel Anti‐Tremor Orthosis: A Randomized Crossover Trial
Source: Mov Disord. 2025 Jan 21;40(3):445–55. doi: 10.1002/mds.30082 (PMC11926495; doi:10.1002/mds.30082)
Supplement: Supplementary file 2 — Table S1. Extended patient demographics. [file MDS-40-445-s003.pdf]

EXTENDED PATIENT DEMOGRAPHICS

SUPPLEMENTARY TABLE S1: PATIENT DEMOGRAPHICS EXTENDED

ID	Sex	Age	Age at tremor onset	Hand that is most affected by tremor	Hand used for ADL tasks	Hand used for writing	Arm with orthosis	Medication use
23	F	82	40	B	R	R	L	None
17	F	78	25	B	R	R	R	Topiramate
7	F	45	10	R	R	R	R	Primidone/Propranolol
12	F	71	60	R	R	R	L	None
5	F	90	65	B	R	R	R	None
3	F	71	63	L	R	R	L	None
16	F	64	59	L	R	R	L	None
2	F	83	76	B	L	R	L	Propranolol
20	M	64	60	R	R	R	R	Propranolol
19	F	78	73	R	R	R	R	None
4	M	75	-	L	R	R	R	Mysoline
14	M	67	50	R	L	R	R	None
1	M	73	60	L	R	R	L	Primidone
22	F	50	14	B	R	R	R	None
9	M	68	47	B	R	R	L	None
13	M	63	16	B	R	R	L	None
6	M	76	45	L	L	L	L	Propanolol/Mysoline
10	F	74	15	L	R	R	L	None
0	M	85	80	B	R	R	R	Gabapentin
21	F	59	10	L	L	L	L	None
18	M	73	48	B	L	R	R	Primidone (ace)
8	M	68	66	L	R	R	L	None
15	M	71	19	R	L	L	R	Propranolol
11	M	80	58	B	R	R	R	Primidone
