# Supplementary material for: Essential Tremor Suppression with a Novel Anti‐Tremor Orthosis: A Randomized Crossover Trial
Source: Mov Disord. 2025 Jan 21;40(3):445–55. doi: 10.1002/mds.30082 (PMC11926495; doi:10.1002/mds.30082)
Supplement: Supplementary file 3 — Figure S2. Example data of accelerometry readings. [file MDS-40-445-s001.pdf]

EXAMPLE DATA OF ACCELEROMETRY READINGS

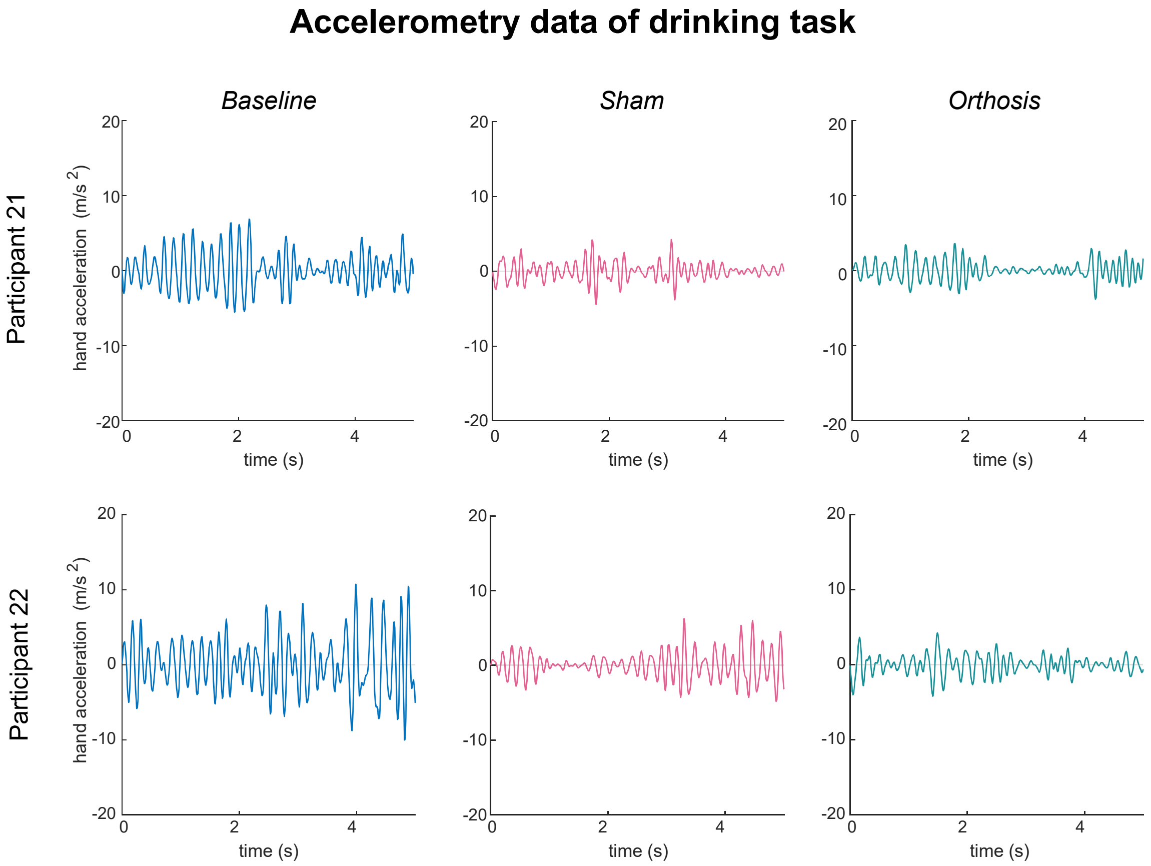

Supplementary Figure S2: Time traces of 5 seconds of band-passed (3-12HZ) hand accelerometry data for the drinking task. Top: participant 21 who scored average on TETRAS reduction; Bottom: participant 22 who scored high in TETRAS reduction. Left: baseline, center: sham and right: orthosis.
