# Supplementary material for: Essential Tremor Suppression with a Novel Anti‐Tremor Orthosis: A Randomized Crossover Trial
Source: Mov Disord. 2025 Jan 21;40(3):445–55. doi: 10.1002/mds.30082 (PMC11926495; doi:10.1002/mds.30082)
Supplement: Supplementary file 4 — Figure S3. TETRAS baseline score VS. TETRAS reduction. [file MDS-40-445-s002.pdf]

TETRAS BASELINE SCORE VS. TETRAS REDUCTION

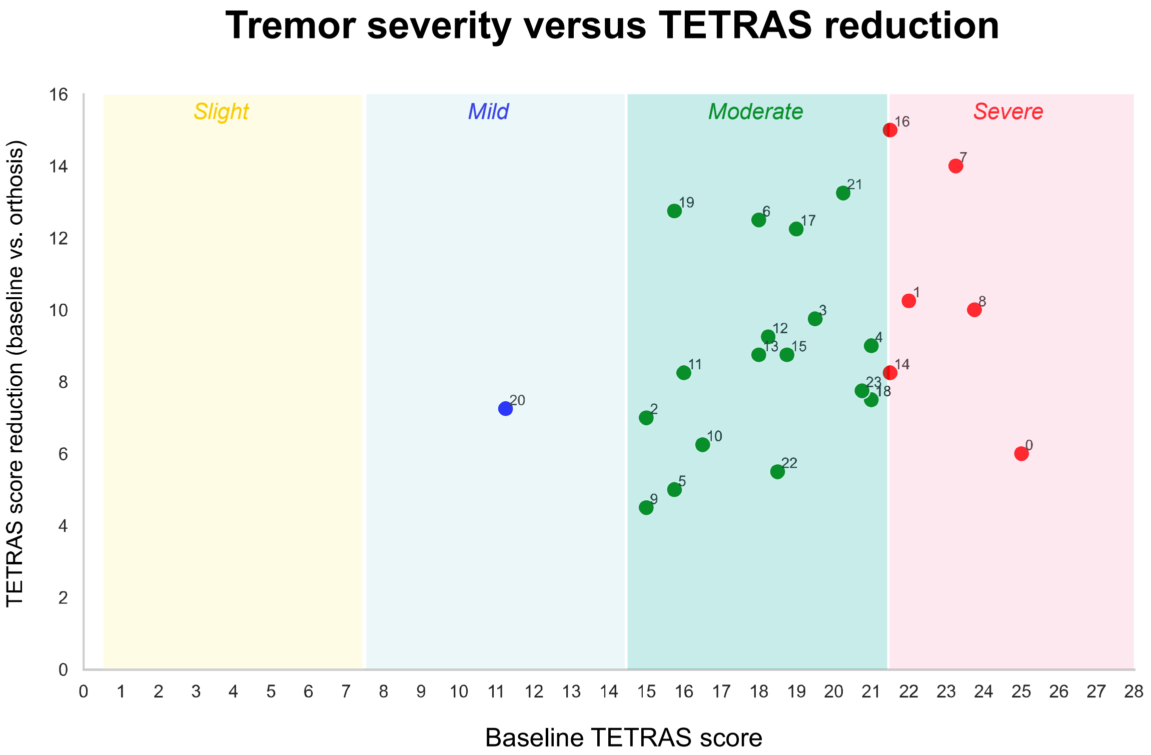

Supplementary Figure S3: Baseline TETRAS against TETRAS Reduction with the orthosis. Mapping of TETRAS scores (max score 28) to tremor severity based on Isaacson (2020) et al.: 0 None, 1-7 Slight, 8-14 Mild, 15-21 Moderate, 22-28 Severe. P20 was included with a baseline TETRAS score of 14, but after averaging of TETRAS scores with the second rater, his average baseline score reduced to below the inclusion threshold.
